# Mitochondrial Cell Death Control in Familial Parkinson Disease

**DOI:** 10.1371/journal.pbio.0050206

**Published:** 2007-07-17

**Authors:** Guido Kroemer, Klas Blomgren

## Abstract

Many sporadic cases of Parkinsons disease have mutations in the PINK protein kinase, whose substrate is now revealed to be a protein that protects mitochondria from oxidative stress.

Parkinson disease (PD) is the most frequent neurodegenerative disorder, affecting about 1% of people over 50 years old. It is caused by the progressive loss of dopaminergic (DA) neurons, accompanied by the accumulation of Lewy bodies, which are abnormal structures inside nerve cells that contain proteins such as a-synuclein, Parkin, and components of the ubiquitin proteasomal pathway (a cellular-degradation pathway). Patients are usually treated with levodopa, and although the drug initially improves motor symptoms, many patients later develop a range of abnormal or uncontrolled muscle movements, called dyskinesias. More than 90% of PD cases are sporadic, but rare genetic forms may yield invaluable information on the pathogenesis of both familial and spontaneous PD.

After mutations affecting Parkin, mutations in PINK1 (PTEN-induced putative kinase 1) are the second-most common cause of autosomal recessive PD (where both parents must contribute a defective gene for PD to arise in the offspring). Point or truncation mutations in PINK1 produce PD with a broad phenotypic spectrum, from early-onset with atypical features to typical late-onset PD. Pathogenic PINK1 mutations—of which about 20 have been identified—annihilate or reduce the kinase activity of the protein. A study by Julia W. Pridgeon (Department of Pharmacology, Emory University School of Medicine, Atlanta, Georgia, United States) and colleages published in this issue of *PLoS Biology* shows that the failure of PINK1 to phosphorylate one particular substrate, TRAP1, can sensitize cells to the lethal effects of reactive oxygen species.

## A Mitochondrial Role in PD

Chemicals that inhibit the mitochondrial electron transport complex I or that elicit production of reactive oxygen species can induce PD in humans, suggesting that mitochondria, which not only produce cellular energy but also control cell death, play a major role in human PD [1]. For both neurons and non-neuronal cells, mitochondrial membranes are the battleground on which opposing signals combat to seal the cell's fate by mitochondrial membrane permeabilization (MMP). Factors that regulate MMP, include the pro- and anti-apoptotic members of the Bcl-2 family, proteins from the mitochondrial permeability transition pore complex (PTPC), and a host of interacting partners. Once MMP occurs, hydrolases and activators of caspases are released. The destructive action of these enzymes, as well as the cessation of the vital bioenergetic and redox functions of mitochondria, finally cause cell death, meaning that mitochondria coordinate the ultimate stage of cellular demise [2].

PINK1 localizes to mitochondria, and experiments in the fruit fly (Drosophila melanogaster) show that PINK1 and DJ1 (another protein that when mutated causes a genetic form of PD) act together to prevent neurodegeneration. PINK1 loss-of-function mutant phenotypes can be rescued by antioxidants; overexpression of Parkin (a ubiquitin E3 ligase) as well as transgenic expression of the anti-apoptotic protein Bcl-2— one of the most potent inhibitors of MMP [3–6]—indicate the existence of a pathway that links most of the proteins that when defective cause PD. Hence, the study of the molecular mechanisms through which PINK1 regulates cell death is likely to yield important clues to the pathogenesis of familial, and perhaps sporadic, PD.

Previously it had been shown that overexpression of PINK1 protects cells from apoptosis induced by the general tyrosine kinase inhibitor staurosporine [7,8] and that pathogenic mutations of PINK1 that inactivate its serine/threonine kinase activity [9] do not protect against cell death, implying that its kinase activity is indispensable for this effect. However, it has not been known which substrates phosphorylated by PINK1 would account for its capacity to inhibit lethal MMP.

## PINK Mutations Place Mitochondria at Risk

**Figure 1:**
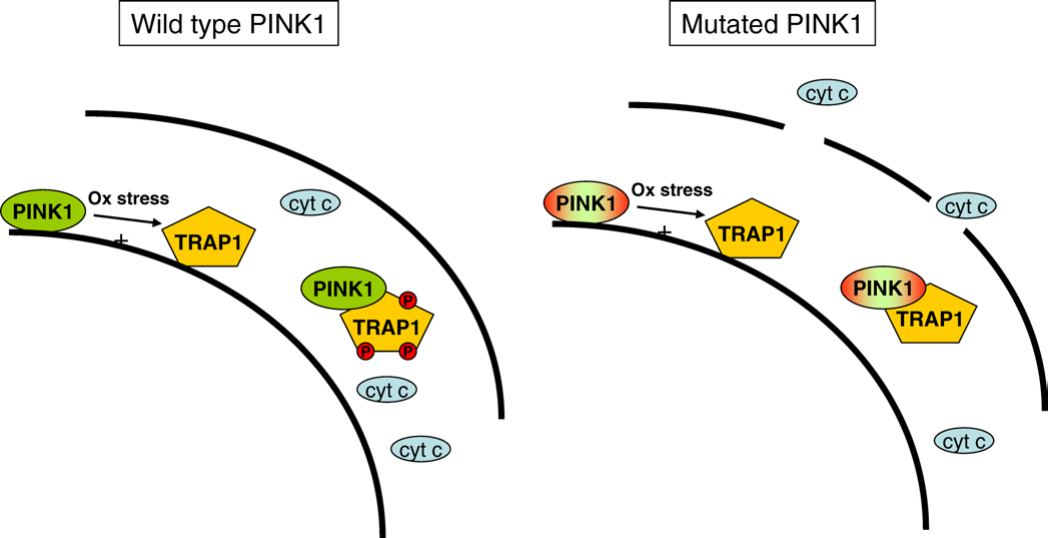
A Mechanism through which PINK1 Mutations under Conditions of Increased Oxidative Stress May Sensitize Dopaminergic Neurons to Cytochrome c Release and Subsequent Cell Death, Eventually Causing PD

In this issue of *PLoS Biology*, Pridgeon and coworkers show that the PINK1 mutations that cause PD (such as PINK1 G309D, L347P, and W437X) have a reduced capacity to phosphorylate TRAP1 and to protect mitochondria and cells against the fatal consequences of oxidative stress (Figure 1). This is a breakthrough in our understanding of the mechanisms underlying inherited PD linked to PINK1 mutations, and it has implications where there is increased susceptibility to oxidative stress, such as in the immature brain [10].

The work by Pridgeon et al. suggests avenues for further exploration. Does PINK1 have other molecular targets in mitochondria? Pridgeon et al. found at least ten proteins that could bind to recombinant PINK1 in vitro—could these too be targets for PINK 1? It is intriguing that some PINK1 mutations cause autosomal recessive PD, yet some cases of late-onset PD are associated with heterozygous mutations in PINK1 [11]. It is possible that a complete defect in PINK1 kinase due to mutation of both *PINK1* alleles would cause early-onset autosomal recessive PD, yet a partial defect in PINK1 kinase would contribute to late-onset autosomal dominant PD.

Future investigations must address the question as to how phosphorylated TRAP1 prevents the release of cytochrome c from mitochondria. Pridgeon et al. clearly show that it was not a question of TRAP1 sequestering of cytochrome c. It would be interesting to know whether phosphorylated TRAP1 impairs MMP and, if so, through what mechanisms. MMP can be triggered in many different ways and TRAP1 is a member of the HSP90 family of heat shock proteins, which are well known for their multiple cytoprotective effects, such as the removal of misfolded proteins or neutralization of pro-apoptotic proteins [12]. Could this be how TRAP1 prevents cell death?

Interestingly, TRAP1 has nonmitochondrial functions that may be regulated by factors other than PINK1. Indeed, the abbreviation stands for “tumor necrosis factor receptor–associated protein 1”. TRAP1 chaperones the retinoblastoma protein, thereby affecting cell-cycle regulation [13]. TRAP1 is upregulated by the oncogene *c-Myc* [14] and down-regulated in HIV-1–infected cells [15]. This latter process may contribute to HIV-1–induced MMP and apoptosis. Moreover, TRAP1 is suppressed by the HSP90 inhibitor geldanamycin [16], suggesting that at least part of the chemosensitizing effects of geldanamycin and its analogs might be attributed to an effect on TRAP1. Hence, TRAP1 may be involved in the general regulation of apoptosis, outside of the central nervous system.

It remains a mystery why the impaired phosphorylation of TRAP1 by mutated PINK1 induces the specific DA neurodegeneration seen in PD. PINK1 is not limited to DA neurons of the substantia nigra, but is expressed throughout the human brain in all cell types, as well as outside the brain, and the same applies to TRAP1 [17]. PINK1 was detected in a proportion of Lewy bodies both in sporadic PD and in PD cases associated with heterozygous mutations in the PINK1 gene, which are clinically and pathologically indistinguishable from the sporadic cases. Interestingly, PINK1 was absent from other protein-filled intracellular structures (called inclusions), such as cortical Lewy bodies, in neurofibrillary tangles in Alzheimer disease, in progressive supranuclear palsy and corticobasal degeneration, and in the glial and neuronal alpha-synuclein positive inclusions in multiple system atrophy [17]. These findings point to specific contributions of PINK1 to PD that remain to be explored, yet they do not explain why PINK1 mutations compromise the survival of DA neurons while leaving other cell types unaffected.

A second integral feature of PD pathogenesis is oxidative stress. Postmortem investigations have consistently shown that oxidative stress is a hallmark of the damaged substantia nigra from PD patients. The preferential loss of nigral neurons in PD has been attributed to the highly oxidative intracellular environment in dopaminergic neurons [18]. Oxidation and degradation of the labile transmitter dopamine may induce oxidative stress, proteolytic dysfunction, and mitochondrial defects, and this may be the underlying reason for the selective vulnerability of DA neurons [19]. Oxidative stress is intimately linked to other aspects of neurodegeneration, such as mitochondrial dysfunction, which may make it difficult to determine whether oxidative stress leads to, or is a consequence of, mitochondrial dysfunction. Inhibition of complex I results in enhanced production of ROS, and increased ROS levels may in turn inhibit complex I, resulting in a vicious cycle of oxidative stress and impaired mitochondrial respiration [20]. Furthermore, the toxicity of mitochondrial poisons such as 1-methyl-4-phenylpyridium ions (MPP^+^) and rotenone is most pronounced in DA neurons, even though they inhibit complex I throughout the brain, suggesting that DA neurons are intrinsically sensitive to complex I defects [20]. Also, complex I inhibitors such as rotenone have been shown to reduce proteasomal activity through ATP depletion, leading to increased toxicity in neurons with a compromised proteasome, thereby constituting a link between mitochondrial activity, proteasomal insufficiency, impaired clearance of misfolded proteins, and cell death [21].

As a result, deficient PINK1 function might selectively affect DA neurons, because DA neurons constitute the cell type that is most susceptible to ROS attack in the human body. Not withstanding this explanation, impaired PINK1 function might also render other neurons more susceptible to oxidative stress, suggesting that other neurodegenerative diseases (e.g., Alzheimer disease) might progress at a faster rate when PINK1 is mutated. Similarly, acute brain injuries (e.g., stroke or trauma) could be exacerbated in the absence of intact PINK1. These issues require urgent clarification because they might affect the clinical management of neurological diseases well beyond that of PD.

## Next Steps

The future will tell whether the functional exploration of PINK1 might furnish new targets for therapy. Theoretically, inhibitors of the phosphatase that dephosphorylates TRAP1 might have a neuroprotective effect on DA neurons Based on these speculations, we are cautiously optimistic that small-molecule inhibitors of kinases may become useful for the prevention or treatment of PD. 3

## Abbreviations

DA: dopaminergic
MMP: mitochondrial membrane permeabilization
PINK1: PTEN-induced putative kinase 1
PD: Parkinson disease
